# Combination of miR159 Mimics and Irinotecan Utilizing Lipid Nanoparticles for Enhanced Treatment of Colorectal Cancer

**DOI:** 10.3390/pharmaceutics16040570

**Published:** 2024-04-22

**Authors:** Rulei Yang, Yiran Liu, Ning Yang, Tian Zhang, Jiazhen Hou, Zongyan He, Yutong Wang, Xujie Sun, Jingshan Shen, Hualiang Jiang, Yuanchao Xie, Tianqun Lang

**Affiliations:** 1 School of Chinese Materia Medica, Nanjing University of Chinese Medicine, Nanjing 210023, China; yangrulei@simm.ac.cn (R.Y.); shenjingshan@simm.ac.cn (J.S.); hljiang@simm.ac.cn (H.J.); 2 State Key Laboratory of Drug Research, Shanghai Institute of Materia Medica, Chinese Academy of Sciences, Shanghai 201203, China; sunxujie@simm.ac.cn; 3 Vigonvita Life Sciences Co., Ltd., Suzhou 215125, China; 4 Lingang Laboratory, Shanghai 200031, China; liuyr@lglab.ac.cn (Y.L.); yangning@lglab.ac.cn (N.Y.); zhangtian@lglab.ac.cn (T.Z.); houjiazhen0380@lglab.ac.cn (J.H.); hezongyan0379@lglab.ac.cn (Z.H.); wangyutong@lglab.ac.cn (Y.W.)

**Keywords:** colorectal cancer, plant-derived miR159, irinotecan, lipid nanoparticle

## Abstract

Colorectal cancer (CRC) ranks as the third most prevalent global malignancy, marked by significant metastasis and post-surgical recurrence, posing formidable challenges to treatment efficacy. The integration of oligonucleotides with chemotherapeutic drugs emerges as a promising strategy for synergistic CRC therapy. The nanoformulation, lipid nanoparticle (LNP), presents the capability to achieve co-delivery of oligonucleotides and chemotherapeutic drugs for cancer therapy. In this study, we constructed lipid nanoparticles, termed as LNP-I-V by microfluidics to co-deliver oligonucleotides miR159 mimics (VDX05001SI) and irinotecan (IRT), demonstrating effective treatment of CRC both in vitro and in vivo. The LNP-I-V exhibited a particle size of 118.67 ± 1.27 nm, ensuring excellent stability and targeting delivery to tumor tissues, where it was internalized and escaped from the endosome with a pH-sensitive profile. Ultimately, LNP-I-V significantly inhibited CRC growth, extended the survival of tumor-bearing mice, and displayed favorable safety profiles. Thus, LNP-I-V held promise as an innovative platform to combine gene therapy and chemotherapy for improving CRC treatment.

## 1. Introduction

Colorectal cancer (CRC) stands as the third most prevalent malignancy globally, ranking fourth in lethality, with an upward trend in recent years. Various risk factors contribute to CRC, encompassing lifestyle, dietary habits, obesity, age, inflammatory bowel disease or CRC history, and family predisposition [1]. Although conventional treatments involving surgery, radiotherapy, and chemotherapy have been applied for clinical treatment, CRC prognosis remains unfavorable, marked by high metastasis and post-surgical recurrence, resulting in a less than 20% 5-year survival rate [2,3].

Nowadays, chemotherapy is still the primary treatment approach following surgical resection of colorectal cancer [4]. Irinotecan (IRT), an FDA-approved topoisomerase I inhibitor derived from the Chinese tree *Camptotheca acuminate*, is a first-line chemotherapeutic drug for metastatic colon or rectal carcinoma [5,6]. However, challenges such as side effects, multidrug resistance, low stability, and short half-life hinder its broad application. An applicable drug delivery system is crucial for optimizing IRT’s anti-tumor effectiveness [7,8,9].

Oligonucleotides including antisense oligonucleotides (ASO), siRNA, shRNA, and miRNA, hold the promise of effectively treating a diverse range of diseases typically associated with undruggable proteins, thereby transcending the limitations imposed by traditional therapies and presenting expansive application prospects. Oligonucleotides, as synthetic nucleic acid polymers, are versatile tools for modulating gene expression through various mechanisms, including targeting pre-mRNA, mRNA, or non-coding RNA, resulting in degradation, modulation of splicing events, or inhibition of protein translation [10,11,12]. More importantly, oligonucleotides have the potential to intricately regulate key processes within tumor cells, encompassing pivotal aspects such as proliferation, differentiation, apoptosis, angiogenesis, metastasis, and drug resistance [13,14,15]. In recent years, miRNA profiling and deep sequencing have provided direct evidence of the relationship between miRNA expression and cancer dysregulation, emphasizing their role in tumor classification, diagnosis, and prognosis [16,17]. Thus, the nucleic acid interference drug is a highly promising therapeutic approach for effectively treating tumors [18,19,20]. A plant-derived miRNA, miR159, identified in *Arabidopsis thaliana*, *Glycine max*, and *broccoli*, inhibits tumor proliferation by targeting TCF7, a Wnt signaling transcription factor. However, the effectiveness and specificity of treatment solely relying on miR159 would be hindered by the heterogeneity of tumor cells. In addition, miR159 demonstrates instability, and it encounters obstacles like endosome escape upon cellular entry. These factors severely limit the anti-tumor applications of plant-derived miRNAs like miR159 [21].

Lipid nanoparticles (LNPs), composed of cationic lipids, helper lipids, cholesterol, and PEG-lipids, stand out as an immensely promising delivery system with substantial potential for both research and clinical applications. LNPs are capable of encapsulating various therapeutic agents, including nucleic acids, small molecules, and monoclonal antibodies, enabling a diverse range of applications. LNPs present distinct advantages in enhancing the pharmacokinetic properties and tissue distribution of therapeutic agents. Whether derived from natural or synthetic lipids, LNPs showcase outstanding biocompatibility and degradability [22,23]. In addition, LNPs also possess the ability to co-deliver multiple drugs and protect nucleic acid drugs from degradation. Li et al. employed LNPs co-loaded with quercetin and oligonucleotides miR150 for targeted therapy against choroidal neovascularization, demonstrating impressive drug-loading capability and specific delivery of both agents, which resulted in the effective suppression of choroidal angiogenesis and a reduction in choroidal neovascularization lesion volume in vivo [24]. Leveraging the enhanced permeability and retention (EPR) effect, LNPs of nanoscale facilitate tumor targeting capacity [25,26,27]. Pan et al. co-loaded stearic acid-doped LNPs with ovalbumin-coding mRNA and TLR4 agonists to develop a potent tumor vaccine. The tumor vaccine enabled tissue-specific mRNA expression in the spleen upon intravenous injection and enhanced adjuvant activity by activating multiple TLRs, thereby promoting Th1 immune responses [28]. Therefore, employing LNPs for the co-delivery of IRT and plant-derived miRNAs holds promise in addressing the issues mentioned above.

**Figure 1:**
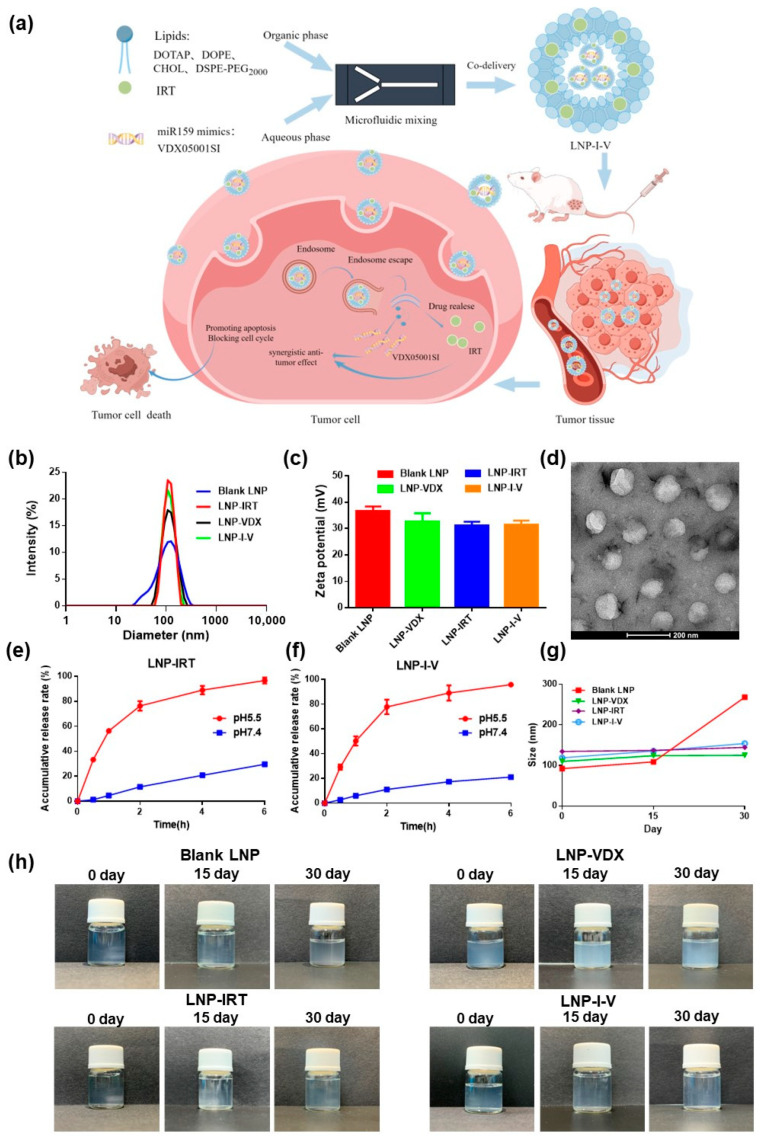
Characterization of LNP formulations. (**a**) Schematic illustration of LNP-I-V for colorectal cancer therapy. By Figdraw. (**b**,**c**) The size (**b**) and zeta potential (**c**) of LNPs. Data are presented as the mean ± SD (*n* = 3). (**d**) The TEM images of LNP-I-V. (**e**,**f**) Release profiles of IRT from LNP-IRT (**e**) and LNP I-V (**f**) in different media. Data are presented as the mean ± SD (*n* = 3). (**g**) The particle sizes of LNPs at 0, 15 and 30 days. Data are presented as the mean ± SD (*n* = 3). (**h**). Solution images of LNP formulations at 0, 15, and 30 days.

To improve the CRC treatment, in this study, we utilized microfluidic technology to construct LNPs for co-delivering IRT and plant-derived miR159 mimics (VDX05001SI, VDX) (Figure 1a). The LNP co-loaded IRT and VDX, termed as LNP-I-V, played a crucial role in preventing drugs from premature clearance upon entering the systemic circulation. LNP-I-V targeting delivered to tumor tissues and internalized by tumor cells. As the vital component of LNP, the cationic lipid DOTAP facilitated endosome escape, leading to the release of IRT and VDX. The synergistic action of oligonucleotides and chemotherapeutic drugs effectively caused tumor cell death by promoting apoptosis and blocking cell cycle. Consequently, LNP-I-V provided a potential strategy for enhancing the therapeutic outcomes of colorectal cancer by integrating chemotherapy and gene therapy.

## 2. Materials and Methods

### 2.1. Materials

The miR159 mimic (VDX05001SI, VDX) with the sense sequence 5′-UUUGGAUUGAAGGGAGCUCUmA-3 (“m” denotes 2′-O-methylation) and anti-sense sequence 5′-GAGCUCCUUGAAGUCCAAUUmG was synthesized by Vigonvita Life Science Co., Ltd. (Suzhou, China). Irinotecan hydrochloride, RPMI 1640 medium, fetal bovine serum (FBS), coumarin-6 (C6), Penicillin-Streptomycin and 1,1′-dioctadecyl-3,3,3,3′-tetramethylindotricarbocyanine iodide (DiR) were purchased from MeilunBio^®^ (Dalian, China). Lipid components, including 1,2-dioleoyl-3-trimethylammonium-propane chloride (DOTAP), N-(Carbonyl-methoxypolyethylene glycol 2000)-1,2-distearoyl-sn-glycerol-3-phosphoethanolamine, sodium salt (DSPE-mPEG_2000_), and 1,2-dioleoyl-sn-glycero-3-phosphoethanolamine (DOPE) were obtained from AVT Pharmaceutical Tech Co., Ltd. (Shanghai, China). Cholesterol was obtained from Acmec Biochemical (Shanghai, China). Dialysis bags with molecular weight cut-off (MWCO) of 2 kDa and 3.5 kDa were purchased from Ruida Henghui Technology Development Co., Ltd. (Beijing, China). Cell Counting Kit-8 (CCK8), Annexin V-FITC/PI Kit, cell nuclei dye Hoechst 33342, and endosome/lysosome dye Lysotracker Red were purchased from Yeasen Biotechnology Co., Ltd. (Shanghai, China). Quanti-iT RiboGreen and trypan blue were obtained from Thermo Fisher (Eugene, OR, USA). Uranium acetate for transmission electron microscopy test was obtained from Zhongjingkeyi Technology Co., Ltd. (Beijing, China). Other reagents and materials were got from Sinopharm Chemical Reagent Co. Ltd. (Shanghai, China). Ultrapure water was produced by the water purification machine (Thermo Fisher, Atvidaberg, Sweden).

### 2.2. Cell Culture

The murine colon cancer cell line CT26 was purchased from MeilunBio^®^ (Dalian, China). The CT26 cells were cultured in RPMI 1640 medium supplemented with 10% FBS and 1% Penicillin-Streptomycin at 37 °C with 5% CO_2_ in the incubator (Thermo Fisher, Marietta, OH, USA).

### 2.3. Animals

Female Balb/c mice aged 4–6 weeks were procured from Shanghai Shengchang Biotechnology Co., Ltd. (Shanghai, China), and were housed in a Specific Pathogen-Free (SPF) grade environment. All the experiments involving animals were conducted under the guideline approved by the Institutional Animal Care and Use Committee (IACUC) of Lingang Laboratory.

### 2.4. Preparation and Characterization of LNP-I-V

The LNP-I-V was prepared using a modified microfluidic method previously outlined for siRNA [29,30,31]. In summary, lipids, including DOTAP, DOPE, cholesterol, and DSPE-mPEG_2000_ in molar ratios of 50:10:38.5:1.5, were dissolved in ethanol. This lipid solution was then combined with phosphate buffer saline (PBS, pH 7.4) solution containing IRT and VDX (*w*/*w* = 100:1) through standard microfluidic mixing at a volumetric ratio of 1:3 (ethanol/aqueous) and a total flow rate of 3 mL/min. Subsequently, the formulation underwent dialysis against 2 L of PBS (pH 7.4) using a dialysis bag (MWCO = 3.5 kDa). After 16 h, the solution was passed through a 0.22 μm filter and stored at 2~8 °C until use. LNPs only encapsulating VDX (LNP-VDX) or IRT (LNP-IRT), and blank LNPs were prepared following the same procedure. The N/P ratios of LNPs containing VDX were maintained at 6.

The LNPs were comprehensive measured for hydrodynamic size, polydispersity index (PDI), and surface zeta potential using Zetasizer Ultra (Malvern Panalytical Inc., Worcestershire, UK). For dynamic light scattering (DLS) analysis, LNPs were suitably diluted in buffers, and triplicate measurements were conducted to ensure accuracy and reproducibility. Furthermore, the morphology of LNP-I-V was observed through transmission electron microscopy (TEM; Tecnai F20, FEI, Hillsboro, OR, USA).

To quantify the encapsulation efficiency (EE) and concentration of the VDX, a modified Quanti-iT RiboGreen RNA protocol (Quant-iT™, R11491) was employed [32]. For IRT, EE and drug loading (DL) were determined through high-performance liquid chromatography (HPLC; Waters, Milford, MA, USA), with a C18 column and UV detection at 254 nm. The mobile phase consisted of a 75 mM ammonium acetate solution and acetonitrile in a volume ratio of 75:25.

The encapsulation efficiency (EE) was calculated using the formula:

The EE% = (weight of encapsulated IRT/weight of feed IRT) × 100%

Drug loading (DL) was determined using the formula:

DL% = (weight of encapsulated IRT/weight of the total LNP) ×100%

### 2.5. In Vitro Release

The release behavior of IRT from LNPs was investigated using the dialysis method. The LNP-IRT or LNP-I-V within a dialysis bag (MWCO = 2 kD) was incubated in the release medium (PBS with pH 7.4 and acetate buffer solution (ABS) with pH 5.5) at 37 °C in a thermostatic cradle, oscillating at a frequency of 100 rpm. At different time points, the supernatant was collected, and release medium of an equivalent volume was replenished. The concentration of IRT in the supernatant was measured using HPLC, following the previously described procedure. All experiments were conducted in triplicate.

### 2.6. Stability of LNP-I-V

The stability of a series of LNPs were investigated. LNP samples were kept in PBS (pH 7.4) at 4 °C. At predetermined time points (0, 15 and 30 days), the samples were analyzed for solution appearance, hydrodynamic diameter, PDI, and zeta potential. All experiments were conducted in triplicate.

### 2.7. Cell Uptake

The fluorescent dye coumarin-6 (C6)-loaded LNP (LNP-C6) was prepared similarly to LNP-I-V. LNP-C6 and free C6 (50 ng/well) were co-cultured with CT26 cells for 1 h, 2 h, and 4 h, followed by staining with LysoTracker Red for lysosome and Hoechst 33342 for nuclei. Trypan blue (0.4%) was added to the cell well for quenching extracellular fluorescence. Afterwards, the cell samples were washed by PBS and fixed by paraformaldehyde. Lastly, the cell samples on microscope slides were observed by laser scanning confocal microscopy (LSCM; Stellaris 5, Leica, Wetzlar Hessen, Germany).

### 2.8. Cytotoxicity

The cytotoxicity of IRT and VDX05001SI toward CT26 cells was assessed using the CCK-8 cytotoxicity assay. CT26 cells (5 × 10^3^ cells per well) were seeded in 96-well plates overnight. For the evaluation of IRT cytotoxicity on CT26 cells, four sample groups were established for testing: 1. Free IRT; 2. Free IRT + VDX; 3. LNP-IRT; 4. LNP-I-V. All the test groups included eight concentrations of IRT.

For the evaluation of VDX cytotoxicity on CT26 cells, three sample groups were established for testing: 1. Free VDX; 2. LNP-VDX; 3. LNP-I-V. All the test groups included five concentrations of VDX.

After treatment of 24 h, the cell viability was assessed utilizing the CCK-8 assay by the plate reader (Envision, Perkin Elmer, Shelton, CT, USA) and calculated following the product manual.

### 2.9. Apoptosis and Cell Cycle Analysis

To evaluate the ability of various formulations to induce apoptosis in CT26 cells, 10^5^ cells per well were seeded in 6-well plates and allowed to incubate for 24 h. Subsequently, eight groups were established for testing: 1. saline; 2. blank LNP; 3. free VDX; 4. free IRT; 5. free IRT + free VDX; 6. LNP-VDX; 7. LNP-IRT; 8. LNP-I-V. The concentration of IRT was maintained at 20 μM for each IRT-containing group, while the VDX concentration was set at 50 nM for each VDX-containing group. Following the manufacturer’s instructions, cells were treated with Annexin V-FITC and propidium iodide (PI). The percentage of apoptotic cells was determined by flow cytometry (NovoCyte, Agilent, San Diego, CA, USA). All experiments were conducted in triplicate.

For cell cycle detection, CT26 cells (10^5^ per well) were seeded in 6-well plates and allowed to incubate for 24 h. Eight sample groups were established for testing, as apoptosis analysis described. Following treatment, the culture medium was discarded, and cells were trypsinized and collected. The collected cells were then fixed with 70% ethanol and stored at −20 °C for 24 h. After centrifugation, the cells were collected and treated with 0.5 mL RNase A (50 μg/mL) before staining with 25 μL propidium iodide (PI) (1.0 mg/mL) for 30 min at 37 °C. Finally, the cell cycle of each group was measured by flow cytometry. All experiments were conducted in triplicate.

### 2.10. Biodistribution

The LNP loaded with DiR (LNP-DiR) was constructed similarly to LNP-I-V. The CRC model was constructed by subcutaneously injecting 1 × 10^6^ CT26 cells on the right back of Balb/c mice. When tumors grew to 300 mm^3^, the mice model were ready to be measured. 2 groups were set: free DiR and LNP-DiR. After intravenous administration (2 mg/kg DiR), mice were euthanized at 1, 4, 8, and 24 h, respectively. Main organs, comprising heart, liver, spleen, lung, kidney, and tumor tissue were extracted. Imagings of these organs were obtained by the in vivo imaging system (NEWTON, Vilber, Collégien, France), and fluorescence intensity of each tumor was also quantified.

### 2.11. In Vivo Anti-Tumor Effect and Immune Analysis

The Balb/c mice were randomly divided into 6 groups (*n* = 6) and each mouse was injected with 1 × 10^6^ CT26 cells on the back. When the tumor reached approximately 150 mm^3^, saline, blank LNP, free IRT + free VDX, LNP-VDX, LNP-IRT, or LNP-I-V were administered to each group (4 mg/kg IRT, 640 ng/kg VDX) through the tail vein twice a week for three weeks. The tumor volumes (major axis × minor axis^2^/2) and body weight of each mouse were regularly recorded throughout the treatment period. During the treatment period, the survival time of each CT26 tumor-bearing mouse was also investigated. In cases when tumor volumes were over 2000 mm^3^, mice had to sacrifice ahead of treatment plan. The median survival time of each group was calculated by the GraphPad Prism software (Version 6.02). On the 21st day after the first administration, all the mice were euthanized, and the tumors were dissected and photographed. The average tumor weight of each group was recorded to figure out the tumor-inhibition rates (TIR) using the provided equation:

TIR = (1 − W_test_/W_saline_) × 100%

To assess cell apoptosis of tumor tissues, the terminal deoxynucleotidyl transferase-mediated dUTP-biotin nick end labeling (TUNEL) assay was employed. For histological analysis, tumor samples were embedded in paraffin blocks, and 5 μm sections were stained with hematoxylin and eosin (H&E) using conventional methods.

In addition, the immune response caused by LNPs was also researched. The tumor tissues were collected to conducted immunofluorescence sections which were labeled CD4 or CD8 antibody.

All the dried and sealed slices were scanned with a digital pathology scanner (VS200, Olympus, Tokyo, Japan).

### 2.12. Biocompatibility

To evaluate the biocompatibility of LNPs, healthy Balb/c mice (*n* = 3, per group) were administrated various formulations as described in the pharmacodynamics evaluation. On Day 21, pathological changes in main organs were tested after constructing H&E sections. The slices of main organs were scanned with a digital pathology scanner.

### 2.13. Statistical Analysis

Statistical analysis was conducted using GraphPad Prism software (Version 6.02). Unpaired Student’s *t*-tests were employed to compare two groups, assuming a Gaussian distribution. For comparisons involving three or more groups, one-way ANOVA was utilized. Statistical significance was denoted by * for *p* < 0.05, ** for *p* < 0.01, and *** for *p* < 0.001.

## 3. Results

### 3.1. Preparation and Characterization of LNP-I-V

Lipid nanoparticles encapsulating IRT and VDX were prepared by microfluidic mixing technology, and their physicochemical properties were characterized. All LNPs exhibited similar physicochemical characteristics. As the control formulation, particle sizes of blank LNP, LNP-IRT, and LNP-VDX were 92.29 ± 0.57 nm, 133.80 ± 1.61 nm, and 109.90 ± 0.26 nm, with zeta potentials of 36.55 ± 1.83 mV, 31.14 ± 1.45 mV, and 32.76 ± 3.06 mV, respectively. For the LNP co-loading IRT and VDX, LNP-I-V, the mean particle size and zeta potential were 118.67 ± 1.27 nm and 31.39 ± 1.64 mV, whose PDI was 0.171 ± 0.018 (Figure 1b,c). The encapsulation efficiency (EE%) of IRT and VDX were 23.91% and 96.00%, and drug loading (DL%) were 0.50% and 0.0013%, respectively, demonstrating the feasibility of the LNP co-delivering strategy. Transmission electron microscopy (TEM) images further confirmed the morphology and mono-dispersity of LNP-I-V, which was consistent with the results obtained from dynamic light scattering (DLS), indicating a homogeneous dispersion formulation (Figure 1d).

The release profiles of IRT from LNP-IRT and LNP-I-V were evaluated under different pH conditions (pH 5.5 and pH 7.4). According to the drug release curves, IRT exhibited slow release from LNP-IRT and LNP-I-V in PBS (pH 7.4) containing 10% FBS, with 29.62 ± 0.78% and 21.11 ± 0.36% of IRT released from LNPs within 6 h, respectively. The drug release rate was significantly rapid in ABS (pH 5.5), with 76.31 ± 3.11% and 77.91 ± 4.88% of IRT released from LNP-IRT and LNP-I-V within 2 h (Figure 1e,f). The variance in the release rates of LNPs in different pH conditions might be attributed to the pH sensitivity of the ionizable lipid DOTAP contained in LNPs, which ionizes in an acidic environment, thereby enhancing drug release.

The stability of LNPs was analyzed in vitro. After 1 month, the hydrodynamic diameter, PDI, and zeta potential of a series of LNPs did not change obviously (Figure 1g, Table 1). In addition, all the solution properties of LNPs maintained colloidal stability, and no aggregation was observed (Figure 1h). Such data verified that LNP helps maintain the stability of the IRT and VDX over time.

### 3.2. Cell Uptake

**Figure 2:**
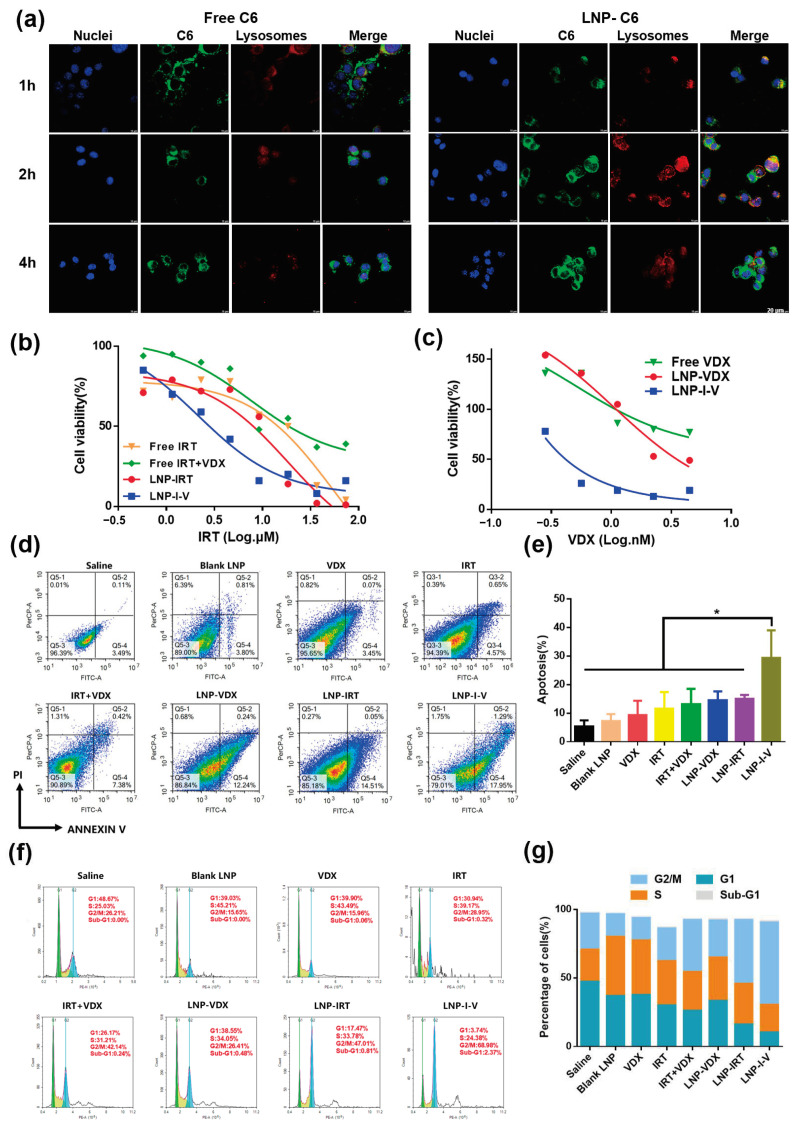
Profiles of LNP-I-V on CT26 cells. (**a**) LSCM images of free C6 and LNP-C6 incubated with CT26 cells (scale bar = 20 μm). (**b**) The cytotoxicity of IRT formulations in CT26 cells. (**c**) The cytotoxicity of VDX in CT26 cells. Data are presented as the mean (*n* = 3) (**d**,**e**) Apoptotic cells (%) caused by different formulations. The density map means that the color from blue to red above refers to how many cells from less to more signal falls on that location. Data are presented as the mean ± SD (*n* = 3). (**f**,**g**) Cell cycle analysis of CT26 cells treated with different formulations by flow cytometry. The histogram uses color to distinguish different cell cycles, with green representing G1 phase, yellow representing S phase, and blue representing G2/M phase. Data are presented as the mean (*n* = 3). * *p* < 0.05.

To observe the uptake of LNPs by tumor cells, the drugs in LNPs were replaced with the fluorescent dye C6. Under laser scanning confocal microscopy (LSCM), as the control group, the free C6 exhibited a dim green fluorescence until 4 h since the hydrophobic free C6 entered the tumor cells via free diffusion (Figure 2a). By contrast, LNP-C6 efficiently entered CT26 colon cancer cells. The co-localization of yellow signals between LNP-C6 (green fluorescence) and lysosomes (red fluorescence) were observed after incubation for 1 h, which indicated endocytosis of LNP-C6 and localization at lysosomes. After 2 h incubation, the green fluorescence gradually diffused in the cells. Then, green fluorescence intensity got stronger in the cytoplasm at 4 h, indicating the endosome escape of cargos. The efficient internalization and drug releasing capacity of LNP guaranteed the IRT and VDX could inhibit tumor growth synergistically.

### 3.3. Cytotoxicity

The CT26 cells were selected as the cell model and the CCK-8 assay was employed to determine the cytotoxicity of IRT and VDX formulations. The cytotoxicity of free IRT + VDX and LNP-IRT was almost same, with IC_50_ values of 12.05 μM and 12.08 μM, respectively, while the IC_50_ value for LNP-I-V was 4.42 μM. In contrast, free IRT exhibited a higher IC_50_ value of 34.62 μM (Figure 2b). The cytotoxicity of VDX formulations, including free VDX, LNP-VDX, and LNP-I-V, gradually increased, with IC50 values of 0.85 nM, 0.36 nM, and 0.12 nM, respectively (Figure 2c). These results indicated that LNP-I-V induced prominent cytotoxicity in CT26 cells. It was also suggested that the synergistic use of VDX might enhance the anti-cancer efficacy of IRT.

### 3.4. Apoptosis and Cell Cycle Analysis

The apoptosis of CT26 cells in different formulation groups was evaluated through the Annexin V/PI staining assay. The flow cytometry results showed that the IRT and/or VDX encapsulated in LNP significantly increased the apoptosis of CT26 cells compared to the free drug (Figure 2d,e). In addition, 29.35 ± 7.89% of CT26 cells treated with LNP-I-V underwent apoptosis, while 14.44 ± 2.63% and 14.96 ± 1.17% cells apoptosis was induced by monotherapy groups of LNP-VDX and LNP-IRT. The results proved that LNP-I-V could cause tumor cell apoptosis obviously by co-delivering IRT and VDX.

The impact of LNP-I-V on the cell cycle status was also investigated in CT26 cells with propidium iodide by flow cytometry. Compared to the saline group and the blank-LNP group, LNP-I-V promoted cells from G1 phase (10.02 ± 5.81%) to G2/M phase (60.17 ± 10.44%) (Figure 2f,g). Meanwhile, a higher G2/M phase and sub-G1 phase were observed in the LNP-I-V group compared to the monotherapy group such as LNP-VDX and LNP-IRT which were consistent with cytotoxicity and apoptosis analysis. Since the most cells in G2/M phase meant that the cells were in a state of slow proliferation or stagnation, the results confirmed that LNP-I-V could effectively inhibit tumor cell growth.

### 3.5. Biodistribution

**Figure 3:**
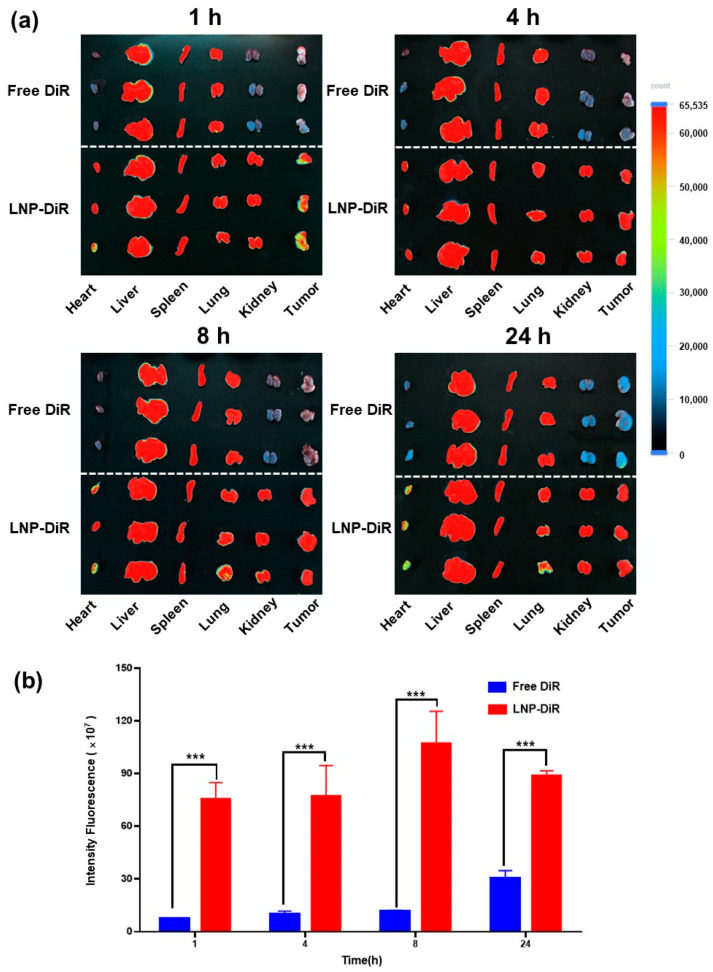
Biodistribution of LNP. (**a**) Ex vivo fluorescence images of main organs/tumor tissues and (**b**) the fluorescence intensity of tumor tissues of CT26 tumor-bearing mice at 1 h, 4 h, 8 h, and 24 h after intravenously administrated with free DiR and LNP-DiR. Data are presented as the mean ± SD (*n* = 3). *** *p* < 0.001.

To investigate the biodistribution profile of LNP-I-V, the fluorescent dye DiR-loaded LNP, LNP-DiR was prepared and the CT26 tumor-bearing mice model was established. As shown by IVIS, free DiR was mainly accumulated in the liver and spleen, barely detected in tumors. In contrast, at the same dose, the intratumoral fluorescence of LNP-DiR was obvious and the fluorescence intensity kept at high level until 24 h after injection (Figure 3a). The semi-quantitative analysis also showed that the concentration of DiR in the LNP-DiR group was much higher than that in the free DiR group at each time point (Figure 3b). The phenomenon demonstrated that the LNP with nano size was able to prevent DiR from quick clearance in the circulation and target delivering cargos to the tumor tissues.

### 3.6. In Vivo Anti-Tumor Efficacy

**Figure 4:**
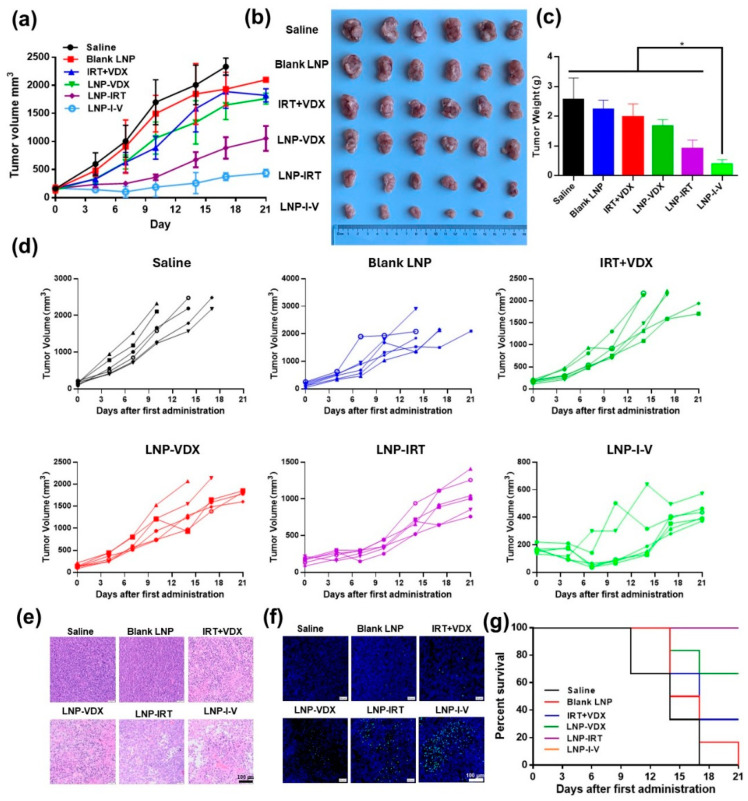
In vivo anti-tumor capacity in the CT26 tumor-bearing mice. (**a**) The average tumor volumes change during the treatment process. (**b**) Images of tumor tissues after 21 days. (**c**) Tumor weights of each group at the end of the therapy process. (**d**) Tumor volume variation during the treatment period of each group. (**e**) Representative H&E sections of tumor (scale bar: 100 μm). (**f**) Immunofluorescence images of tumor sections examined by the TUNEL assay (scale bar: 100 μm). (**g**) Survival curves of CT26 tumor-bearing mice (*n* = 6). Data are presented as the mean ± SD (*n* = 6). * *p* < 0.05.

The anti-tumor efficacy of LNP-I-V was examined on the CT26 tumor xenograft model. Tumor sizes and body weights were recorded during the 21-day treatment period. The saline and blank LNP were not able to control tumor growth, demonstrating that blank LNP as a carrier did not possess the therapeutic effect itself. Free IRT + VDX slightly slowed down the growth of tumors with a tumor-inhibition rate (TIR) of 23.16% owing to the low intra-tumoral drug distribution. Differing from free drug, LNP-VDX and LNP-IRT showed better tumor growth inhibition effects with a TIR of 35.20% and 64.35%, proving that utilizing the LNP as the delivery platform was an effective approach to improving therapeutic efficacy of drugs. Combining the merits of IRT and VDX, LNP-I-V showed the strongest tumor suppression capacity. The TIR of LNP-I-V was 85.17%, which was 3.7, 2.4, and 1.3 times those of free IRT + VDX LNP-VDX, and LNP-IRT, respectively (Figure 4a–d). Meanwhile, the tumors from different groups were collected to conduct histological examination. The H&E and the TUNEL assay showed that there was almost no apoptosis found in tumors from mice administrated with saline and blank LNP. Free IRT + VDX, LNP-VDX, and LNP-IRT caused apoptosis to a certain scale, while LNP-I-V induced cell apoptosis of a big area in tumor tissue sections (Figure 4e,f). It must also be mentioned that the there were no mice of the LNP-I-V group that died or exhibited tumor volumes over 2000 mm^3^ during the treatment period (Figure 4g). This meant that LNP-I-V achieved a significant extension of the survival period of the tumor-bearing mice.

### 3.7. Anti-Tumor Immunity

**Figure 5:**
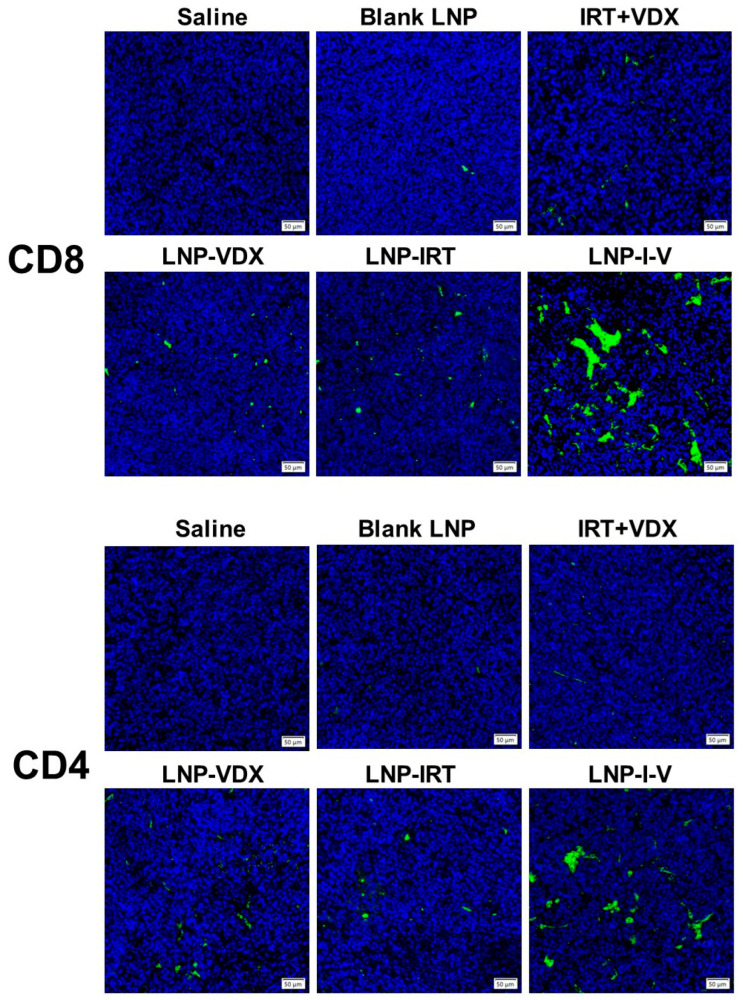
Anti-tumor immune responses in CT26 tumor-bearing mice. The representative immunofluorescence sections of CD8^+^ and CD4^+^ T cells in tumor tissues. (scale bar: 50 μm).

In addition to the increased drug content in tumors, the tumor-inhibition effect of LNP-I-V could be ascribed to its immunomodulating capacity. The adaptive anti-tumor immune response relies primarily on CD4^+^ and CD8^+^ T cells. Immunofluorescence tumor sections from the saline group and blank LNP showed minimal infiltration of CD4^+^ and CD8^+^ T cells, indicating an immunosuppressive tumor microenvironment (Figure 5). In comparison, LNP-I-V induced the infiltration of CD4^+^ and CD8^+^ T cells in the tumor tissues, revealing that the stimulating anti-tumor immunity ability of co-delivering oligonucleotides and chemotherapeutic drugs by LNP.

### 3.8. Biocompatibility

**Figure 6:**
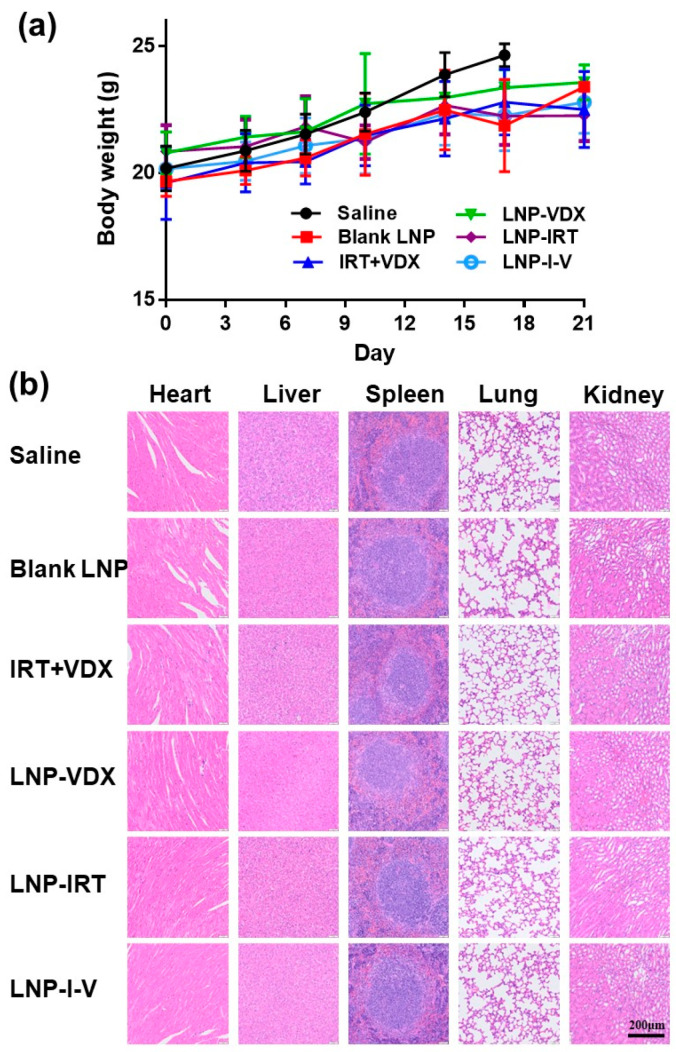
Biosafety evaluation of different formulations. (**a**) The body weight variation in mice during the therapy period. Data are presented as the mean ± SD (*n* = 6). (**b**) Images of H&E-stained tissue sections of main organs in healthy mice (scale bar: 200 μm).

Since the cytotoxic agent IRT was delivered by LNP containing a cationic lipid (DOTAP), the biocompatibility of LNP-I-V was supposed to be evaluated. The mice body weights of LNP-I-V remained stable throughout the therapy period (Figure 6a). In addition, the hematoxylin and eosin (H&E)-stained sections of main organs from healthy mice treated with LNP-I-V did not show obvious pathological damage, which were similar to the saline group (Figure 6b). These results demonstrated that LNP-I-V possessed favorable biosafety.

## 4. Discussion

Utilizing nanotechnology, the LNP provides a solution to the challenges posed by the instability of oligonucleotide agents and the insolubility of chemical therapy drugs [33]. As the key components of LNPs, cationic lipids encapsulate the oligonucleotides and assist endosome escape. Helper lipids such as DSPC and DOPE play a crucial role in maintaining the structural integrity of LNPs, promoting their stability, and facilitating effective intracellular payload delivery. PEG-lipid components provide steric stability, preventing particle aggregation, maintaining small diameters, and ensuring long-term colloidal stability. Additionally, cholesterol, a natural component of cell membranes, enhances LNP uptake and provides structural rigidity [34]. The four components work together to optimize the pharmacokinetic profiles, distribution, and cellular uptake of drugs [35]. The in vivo behavior of LNPs is influenced by their physicochemical properties, including size, surface charge, and functional lipid groups [36]. These factors play a crucial role in determining the efficacy of drug delivery systems. The LNP co-delivering IRT and VDX was meticulously prepared by microfluidic methods, ensuring uniform particle size, distribution, and reproducibility in the manufacturing process. To confirm the ratio of IRT to VDX, we constructed a series of LNPs with the ratio of IRT to VDX like 5:1, 10:1, 20:1, 50:1, 100:1 and 200:1. The EE% of IRT and VDX were set as the parameter to evaluate the LNPs. The EE% of IRT was almost 20% in most LNPs other than the one with ratio of 200:1 whose EE% was very low. As for VDX, the EE% was increased with the ratio elevated. In addition, 100:1 meant the high DL% of IRT, which was beneficial for CRC therapy. By integrating DLS results with TEM images, the size of LNP-I-V met the dimensional requirements for effective accumulation at tumor sites through EPR effect. The in vitro results suggested that LNP-I-V with good stability would prevent drug leakage during circulation in the bloodstream, thereby making it suitable for intravenous injection.

The pH-sensitive drug release behavior of LNPs was attributed to the ionizable lipid DOTAP within the formulation. The DOTAP led to ionization in acidic environments which was consistent with the endosome physiological environment. Assisted by the DOTAP, LNP-I-V could attain the sufficient endosome escape and controlled drug release of cytotoxic agents and oligonucleotides. This controlled drug release behavior at pH of 5.5 also prevented systemic drug toxicity.

The free drug mainly accumulated in liver and spleen after systematic administration since there was rich blood flow in the two organs. The nano drug delivery system could target to the tumor tissues owing to the EPR effect. LNP-I-V with nano size effectively prevented the premature release of IRT and VDX in the circulation and delivered cargos to targeting sites, ensuring drug stability and killing tumor cells. Furthermore, the targeting capabilities of LNPs minimized toxicity to normal tissues and contributed to the overall safety of LNP-I-V.

Cancer, characterized by gene expression change, genetic mutations, and genomic instability, poses a significant challenge for effective treatment. While synergistic effects of treating cancer are possible with suitable drug combinations, the rise of multidrug resistance (MDR) has undermined the effectiveness of traditional small molecule combinations due to tumor cells possessing efflux pumps and increased resistance to apoptosis. Fortunately, oligonucleotide-based combination therapy has shown considerable success in treating complex diseases, offering enhanced efficacy as a key advantage [37]. In a study by Shahidi et al., a layer-by-layer liposome was utilized to co-deliver 5-fluorouracil (5-FU), si-KRAS, and miRNA-532-3p for the treatment of CRC. This approach demonstrated high tumor uptake and significant inhibition of tumor growth, without notable changes in body weight or organ toxicity [38]. Similarly, Xu et al. designed nanocarriers for co-delivery of 5-FU and miRNA-34a mimics targeted to CRC cells. The results indicated that this co-delivery strategy not only enhanced the anti-CRC activity of 5-FU by silencing sirt1 expression but also inhibited CRC cell migration by targeting CD44. These findings suggested that co-delivery of 5-FU and miRNA-34a mimics could synergistically suppress tumor growth with minimal side effects [39]. Combination therapy, as demonstrated in this study, proves to be a feasible approach for chemo-gene therapy against colorectal cancer. The LNP-I-V exhibited superior anti-tumor effects compared to single mode treatments, inducing increased apoptosis in tumor cells. The adaptive anti-tumor immune process involves the release of tumor-associated antigens (TAAs), presenting antigens, T cell activation and growth, and the subsequent destroy of tumor cells by immunologic effector cells or their secreted cytokines. IRT and VDX might induce immunogenic cell death, enhancing tumor immunogenicity, and promoting the infiltration of T cells in tumor tissues.

## 5. Conclusions

In summary, a lipid nanoparticle encapsulating the chemotherapeutic drug IRT and the oligonucleotide VDX, termed LNP-I-V, was constructed by microfluidic mixing technology for chemo-gene therapy of colorectal cancer. LNP-I-V increased the drug content in tumors, leveraging the EPR effect. After internalization by CRC cells, LNP-I-V disassembled under the acidic environment of endosomes, causing rapid drug release. Consequently, the combination of IRT and VDX by LNP-I-V effectively inhibited tumor growth without inducing obvious side effects. The survival time of tumor-bearing mice treated with LNP-I-V was also prolonged. LNP-I-V provided a viable delivery system for combating colorectal cancer with clinical translation potential. Moreover, this research emphasizes that plant-derived oligonucleotides can serve as a valuable source for the discovery of original new drugs. More plant-derived oligonucleotides will be developed for cancer treatment in the future. Overall, the findings contribute to advancing the understanding of combinational therapeutic approaches and offer potential avenues for the development of more effective anti-cancer clinical treatments.

## Figures and Tables

**Table 1:** Characterization of LNPs formulations. (*n* = 3, mean ± SD).

Formulations	Day	Size (nm)	PDI	Zeta (mV)
LNP-VDX	0	109.90 ± 0.26	0.101 ± 0.032	32.41 ± 1.23
	15	124.00 ± 1.66	0.090 ± 0.006	35.79 ± 2.24
	30	125.07 ± 1.10	0.086 ± 0.012	36.09 ± 7.13
LNP-IRT	0	133.80 ± 1.61	0.257 ± 0.010	31.14 ± 1.45
	15	137.13 ± 2.84	0.162 ± 0.008	23.38 ± 0.59
	30	144.57 ± 1.08	0.233 ± 0.019	20.09 ± 0.33
LNP-I-V	0	118.67 ± 1.27	0.171 ± 0.018	31.39 ± 1.64
	15	135.47 ± 5.29	0.183 ± 0.060	21.98 ± 0.79
	30	154.10 ± 1.31	0.095 ± 0.043	34.70 ± 1.33
Blank LNP	0	92.29 ± 0.57	0.214 ± 0.014	36.55 ± 1.83
	15	108.93 ± 0.55	0.259 ± 0.002	21.59 ± 1.10
	30	268.23 ± 0.80	0.241 ± 0.025	41.57 ± 0.88

## Data Availability

All data are present within the manuscript.
